# Detection of covert lesions in focal epilepsy using computational analysis of multimodal magnetic resonance imaging data

**DOI:** 10.1111/epi.16836

**Published:** 2021-02-10

**Authors:** Baris Kanber, Sjoerd B. Vos, Jane de Tisi, Tobias C. Wood, Gareth J. Barker, Roman Rodionov, Fahmida Amin Chowdhury, Maria Thom, Daniel C. Alexander, John S. Duncan, Gavin P. Winston

**Affiliations:** ^1^ Centre for Medical Image Computing University College London London UK; ^2^ Department of Clinical and Experimental Epilepsy UCL Queen Square Institute of Neurology London UK; ^3^ MRI Unit Epilepsy Society Chalfont St Peter UK; ^4^ National Institute for Health Research Biomedical Research Centre at University College London and University College London NHS Foundation Trust London UK; ^5^ Neuroradiological Academic Unit UCL Queen Square Institute of Neurology London UK; ^6^ Department of Neuroimaging King’s College London London UK; ^7^ Division of Neuropathology The National Hospital for Neurology and Neurosurgery London UK; ^8^ Department of Medicine Division of Neurology Queen's University Kingston Canada

**Keywords:** covert, epilepsy, lesion, MRI, stereoelectroencephalography

## Abstract

**Objective:**

To compare the location of suspect lesions detected by computational analysis of multimodal magnetic resonance imaging data with areas of seizure onset, early propagation, and interictal epileptiform discharges (IEDs) identified with stereoelectroencephalography (SEEG) in a cohort of patients with medically refractory focal epilepsy and radiologically normal magnetic resonance imaging (MRI) scans.

**Methods:**

We developed a method of lesion detection using computational analysis of multimodal MRI data in a cohort of 62 control subjects, and 42 patients with focal epilepsy and MRI‐visible lesions. We then applied it to detect covert lesions in 27 focal epilepsy patients with radiologically normal MRI scans, comparing our findings with the areas of seizure onset, early propagation, and IEDs identified at SEEG.

**Results:**

Seizure‐onset zones (SoZs) were identified at SEEG in 18 of the 27 patients (67%) with radiologically normal MRI scans. In 11 of these 18 cases (61%), concordant abnormalities were detected by our method. In the remaining seven cases, either early seizure propagation or IEDs were observed within the abnormalities detected, or there were additional areas of imaging abnormalities found by our method that were not sampled at SEEG. In one of the nine patients (11%) in whom SEEG was inconclusive, an abnormality, which may have been involved in seizures, was identified by our method and was not sampled at SEEG.

**Significance:**

Computational analysis of multimodal MRI data revealed covert abnormalities in the majority of patients with refractory focal epilepsy and radiologically normal MRI that co‐located with SEEG defined zones of seizure onset. The method could help identify areas that should be targeted with SEEG when considering epilepsy surgery.

**Key Points**

- Computational analysis of multimodal magnetic resonance imaging (MRI) data can help localize the seizure‐onset zone (SoZ) in patients with medically refractory focal epilepsy and radiologically normal MRI
- Comparison with the areas of seizure onset, early propagation, and interictal epileptiform discharges (IEDs) identified at SEEG represent a direct means of validation
- The method may aid refining the hypothesis for SEEG implantation

## 1 INTRODUCTION

Surgical intervention in medically refractory focal epilepsy is only recommended if the area of the brain responsible for seizures can be reliably localized and safely resected. The ideal clinical scenario is if a lesion, found on magnetic resonance imaging (MRI), is confirmed to be involved in seizures by clinical consensus and the risk‐to‐benefit ratio of resecting the brain area responsible is favorable. In 20%–40% of individuals with medically refractory focal epilepsy, no relevant abnormality is visualized on MRI (“MRI‐negative”).^1^ Other investigations such as scalp video electroencephalography (EEG) can guide lateralization and localization of seizures, but may not, in and of themselves, provide accurate localizing data. MRI‐negative epilepsies are a major clinical challenge^2^ and there would be considerable clinical benefit from being able to detect the epileptogenic brain areas in such cases.

Recent research has shown that subtle imaging abnormalities that are not evident on visual reading exist in many individuals with MRI‐negative epilepsy,^3^, ^4^, ^5^ and appropriate image analysis techniques may identify such covert abnormalities, while attempting to balance sensitivity and specificity.^6^, ^7^, ^8^, ^9^, ^10^, ^11^, ^12^ The reliability of these new methods need to be demonstrated^13^ before consideration of a clinical trial to evaluate whether their inclusion in the clinical workflow results in a reduction in the number of patients that cannot be offered curative surgery, and an increase in postoperative seizure freedom.

Most current computational neuroimaging methods are focused on detection of focal cortical dysplasia (FCD), but numerous other pathologies also need consideration. There is also a need to gain specificity, which may be achieved by combining information provided by multimodality imaging.^14^ Another consideration is the validity of the ground truth lesion localization, which has generally been based on an approximate lesion location determined by a consensus among experts^6^ or inferred from postsurgical scans containing the resection zone.^7^, ^8^, ^15^ The latter typically includes nonlesional tissues and cannot be assumed to contain the actual seizure‐onset zone (SoZ) and the epileptogenic zone (EZ), unless seizure freedom postsurgery can be demonstrated. Furthermore, postsurgical scans may not always be available, as surgery may not be performed in all cases, particularly if the risk‐to‐ benefit ratio of the chances of seizure freedom and of causing new deficits is not attractive.

To address these issues, we have developed a method of lesion localization based on the computational analysis of regional and voxel‐based brain MRI features from multimodal MRI data that is not specific to any type of lesion. Our imaging protocol included T1‐weighted imaging, fluid‐attenuated inversion recovery (FLAIR) imaging, susceptibility weighted imaging, multi‐shell, diffusion‐weighted imaging allowing diffusion tensor imaging (DTI) analysis^16^ and neurite orientation dispersion and density imaging (NODDI)^17^ fitting, and driven equilibrium single‐pulse observation of T1/T2 (DESPOT) imaging providing T1 and proton density (PD) maps.^18^, ^19^, ^20^ These sequences were selected to capture a wide range of pathological imaging changes, including changes in water diffusion and magnetic susceptibility. We made no assumptions as to the localization of the covert lesions and directly compared the location of computationally detected imaging abnormalities with areas of seizure onset, early propagation, and interictal epileptic activity identified by the involvement of stereo‐electroencephalography (SEEG) contacts.

## 2 METHODS

This was a prospective study recruiting patients with medically refractory focal epilepsy undergoing presurgical evaluation at the National Hospital for Neurology and Neurosurgery (NHNN), London, UK, and control subjects. The diagnosis was established by clinical consensus during a multidisciplinary team meeting considering history and seizure semiology, 3 T structural MRI with an epilepsy protocol, prolonged video‐EEG telemetry, and neuropsychology. Additional investigations, where relevant, included ^18^F‐fluorodeoxyglucose (FDG) positron emission tomography (PET) and single‐photon emission computed tomography (SPECT). We included patients with normal MRI on visual reading by neuroradiologists specializing in epilepsy who were recommended for SEEG. The study was approved by the NHNN and University College London (UCL) Queen Square Institute of Neurology Joint Ethics Committee. All subjects gave written, informed consent before participating in the study.

One hundred thirty‐one subjects were recruited: 62 healthy controls (median age 39 years, interquartile range [IQR] 30–50, 22 male) with no history of neurological or psychiatric disease, and 69 patients with medically refractory focal epilepsy (Table 1). Of the 69 patients, 42 were MRI positive (median age 34 years, IQR 29–38, 21 male) with potentially epileptogenic lesions identified upon radiological review of their MRI. Nineteen of the 42 MRI‐positive patients had only hippocampal sclerosis identified on MRI. The imaging diagnosis for the remaining 23 patients included cavernomas, encephalomalacia, dysembryoplastic neuroepithelial tumors (DNETs), FCD, ganglioglioma, and heterotopia. Twenty‐seven of the 69 medically refractory focal epilepsy patients were MRI negative (median age 35 years, IQR 25–39, 17 male) with no relevant lesions found upon neuroradiological assessment and subsequently underwent SEEG (Table S1). Each subject underwent a standard MRI protocol from which relevant quantitative measures were derived (Table S2).

**TABLE 1:** Demographics and clinical summary of the control subjects and the two patient groups

	Control subjects	MRI‐positive patients	MRI‐negative patients
Number of patients	62	42	27
Age^a^	39 [30–50]	34 [29–38]	35 [25–39]
Gender (m/f)	22/40	21/21	17/10
Age at disease onset^a^	Not applicable	15 [6–22]	14 [7–19]
Disease duration^a^	Not applicable	20 [9–29]	18 [11–27]

^a^ Given in years as median [interquartile range].

**FIGURE 1:**
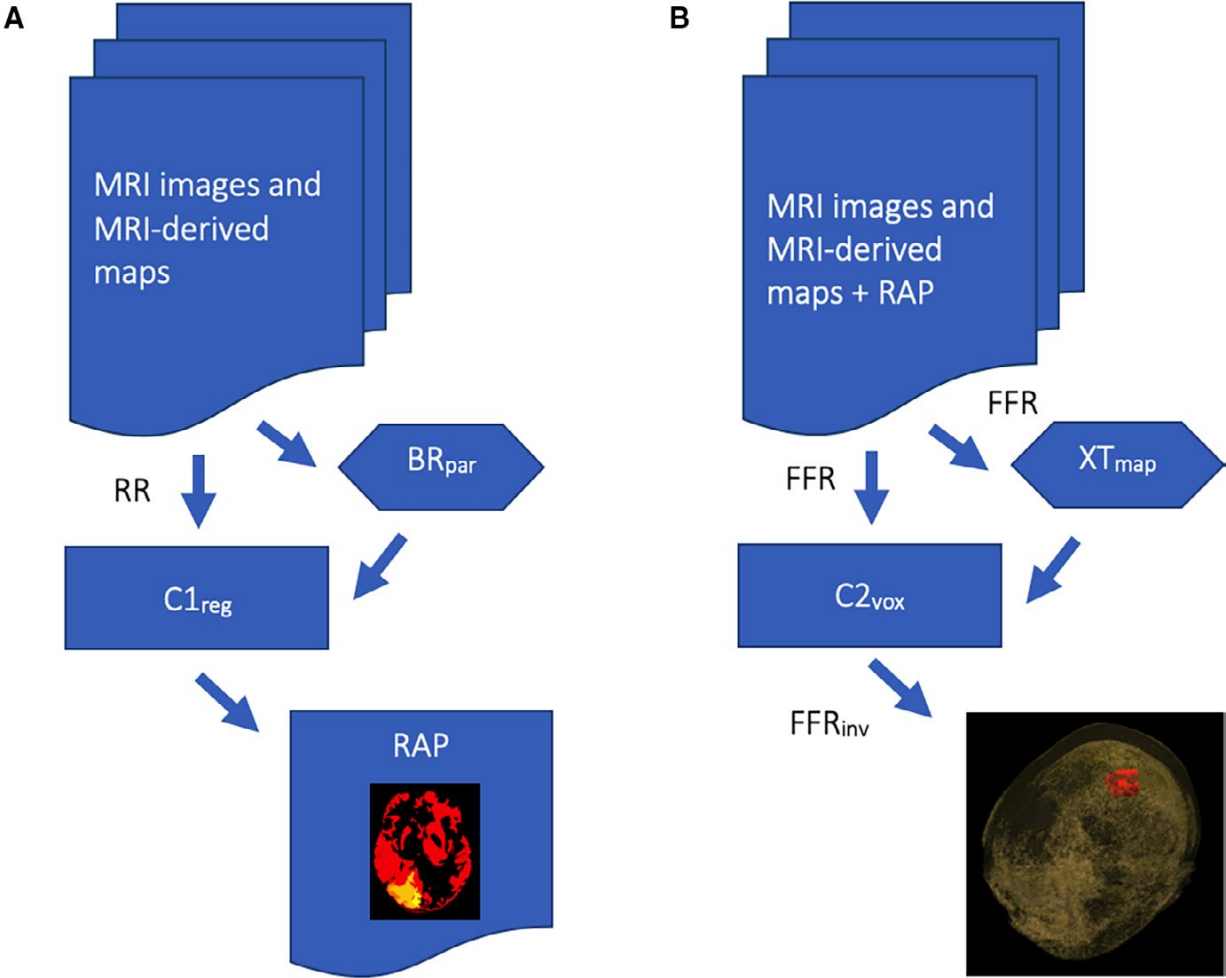
The focal epilepsy lesion detection workflow. (A) MRI images (eg, 3D T1, 3D FLAIR) are used as input to the workflow, alongside MRI‐derived maps (eg, DESPOT T1, NODDI NDI, DTI FA). These images, rigidly registered (RR) to the 3D T1, together with the brain parcellation (BR_par_) obtained from the 3D T1, are used for regional feature extraction and comprise the input to the regional abnormality detector (C1_reg_). The output from this stage is a probabilistic map of regional brain abnormalities (RAP). (B) This output, together with the original input data, are nonrigidly registered to the MNI152 space (FFR), and alongside the cortical thickness map (XT_map_) obtained from the 3D T1 in the same space are used for voxelwise feature extraction and utilised as input to the voxelwise abnormality detector (C2_vox_). An additional input, not shown in the figure, is the GIF parcellation of the MNI152 brain atlas. The output from C2_vox_ is a voxelwise, probabilistic map of brain abnormalities in MNI152 space, which is converted back to the original 3D T1 space by inverse transformation (FFR_inv_) and constitutes the final output of the pipeline

We developed a computational method of lesion detection, comprising two stacked gradient boosting decision tree (GBDT) LightGBM (Microsoft Corporation, Redmond, Washington, USA)^21^ classifiers/regressors. Decision trees are a class of widely used machine learning algorithms, which achieve state‐of‐the‐art performance in many tasks including in lesion and tissue segmentation.^22^, ^23^, ^24^, ^25^, ^26^, ^27^ The first GBDT was a LightGBM regressor (C1_reg_) trained on features of the brain on a per parcellated brain region basis, whereas the second GBDT was a LightGBM classifier (C2_vox_) trained on voxelwise brain features in standard Montreal Neurological Institute 152 (MNI152) space. The workflow is shown in Figure 1.

In the case of the MRI‐positive patients, lesions were outlined by an epileptologist (GPW) using the T1‐weighted and the FLAIR scans (the latter having been rigidly registered to the T1‐weighted scan using NiftyReg^28^) resulting in a binary lesion mask for each MRI‐positive patient. These lesion masks, with empty masks for the control subjects, were used to train and perform the primary validation of C1_reg_ and C2_vox_. We subsequently used the two classifiers/regressors to check for the presence of any covert abnormalities in the MRI‐negative cases. Computationally detected abnormalities were not used to influence the presurgical evaluation of patients including the planning of SEEG.

### 2.1 C1_reg_ (the regional abnormality detector)

**FIGURE 2:**
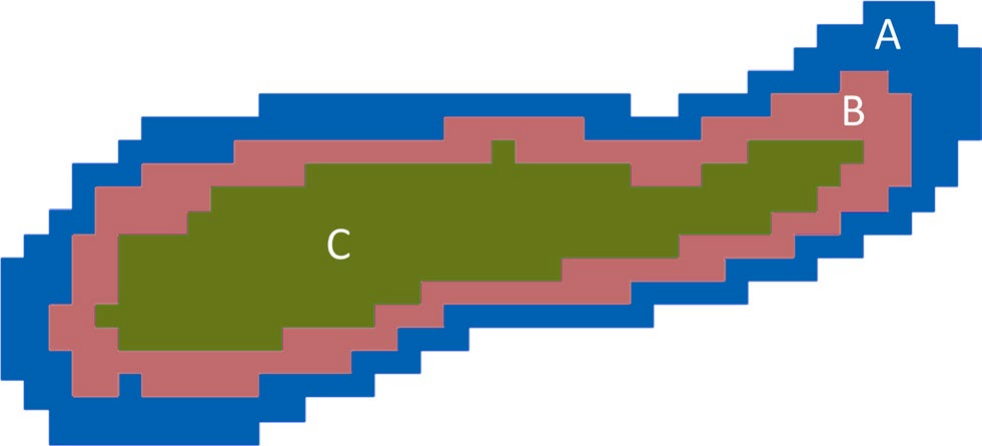
Illustration, in cross‐section, of the extraction of the boundary volumes of a brain region. The brain region shown in this example occupies the volume given by the union of B and C. Volume C is the regional volume deflated by 1 mm around its boundary, whereas the union of A, B, and C is the same volume inflated by 1 mm around the regional boundary. The boundary regions of this brain region are then taken as B, and A, corresponding to a 1 mm thick section of tissue internal to the regional boundary, and a 1 mm thick section of tissue external to the regional boundary, respectively

The regional GBDT regressor was trained to learn what makes a brain region normal or abnormal in terms of its normalized volume, and its appearance on all MRI contrasts and derived maps (Table S2). For this purpose, these MRI contrasts and derived maps were rigidly registered to the T1‐weighted scan using NiftyReg,^28^ and each T1 volume was parcellated into 157 brain regions using GIF.^29^ The characteristics of each brain region were then measured and used as input to the regressor (Table S3). The latter two regional brain properties listed in Table S3 were measured for every available MRI contrast and derived map, not only for the actual volume occupied by each region, but also for the two boundary volumes of each brain region (Figure 2). This was done to assist in detecting pathological regional boundaries.

We trained C1_reg_ to learn a mapping from these inputs to a target value which was set to the percentage of the volume of each brain region marked as being lesional from the manually drawn lesion masks. For example, for a patient whose particular brain region was 10% lesional, in terms of the proportion of the regional volume affected, the C1_reg_ was trained to learn a mapping from the measured characteristics of that brain region to the value 0.1. The same target value for a control subject or a brain region completely outside lesional areas would have been 0.

### 2.2 C2_vox_ (the voxelwise abnormality detector)

The input to the voxelwise abnormality detector was the output from C1_reg_ alongside the following MRI contrasts and derived maps: 3D T1‐weighted scan, 3D FLAIR, NODDI neurite density index (NDI), DTI axial diffusivity (AD), DTI fractional anisotropy (FA), and DTI radial diffusivity (RD) (Table S2). The DESPOT PD, DESPOT T1, and susceptibility weighted scans were not further utilized for C2_vox_, as these contrasts, having been used by C1_reg_, were not found to bring any additional benefit to C2_vox_ in our preliminary investigations. The selected contrasts were all nonrigidly registered to MNI152 space using NiftyReg^28^ (Figure 1). Two additional inputs to C2_vox_ were a map of brain cortical thickness estimated directly in MNI152 space using ANTS^30^, and the GIF parcellation of the MNI152 atlas. The latter was included to provide spatial context to C2_vox_. In other words, this informed C2_vox_ to which region of the MNI152 brain atlas each voxel corresponded.

C2_vox_ was trained to learn a mapping from the signal intensity of each voxel, on each input volume, together with its six immediate neighbors in three dimensions (3D) to a target value of 1 or 0, depending on whether the central voxel fell within an area marked as lesional or nonlesional. For control subjects, all voxels had a target value of 0, indicating the absence of lesions. The predicted lesion mask was subsequently transformed back to the space of the original T1‐weighted scan using inverse transformation.

### 2.3 Validation

For MRI‐positive patients, we used the leave‐one‐out cross‐validation scheme and measured lesion detection performance using the dice score coefficient (DSC). DSC is a measure of spatial overlap and was calculated between the ground truth lesion masks and the output from C2_vox_. For control subjects, we assessed whether any false positive voxels were detected.

For MRI‐negative patients, we made a principled comparison of the output from C2_vox_ and the results of SEEG. A sphere of 10 mm diameter was drawn and visualized in 3D using EpiNav^31^ at the spatial position of each electrode contact that was involved in ictal (seizure onset and early propagation) and inter‐ictal discharges and overlaid on the brain abnormality mask obtained from C2_vox_. The following were then noted by consensus agreement between an epileptologist (JD), a neurophysiologist (FAC), and two senior research fellows (RR and BK): whether the SoZ was within an area marked as abnormal by C2_vox_, whether there was early seizure propagation within the areas marked as abnormal by C2_vox_, whether there were interictal epileptiform discharges within the areas marked as abnormal by C2_vox_, whether there were areas marked as abnormal by C2_vox_ that were not sampled by the SEEG electrodes, and whether there were areas marked as abnormal by C2_vox_ that were sampled by the SEEG electrodes but did not give rise to seizures.^13^

## 3 RESULTS

**FIGURE 3:**
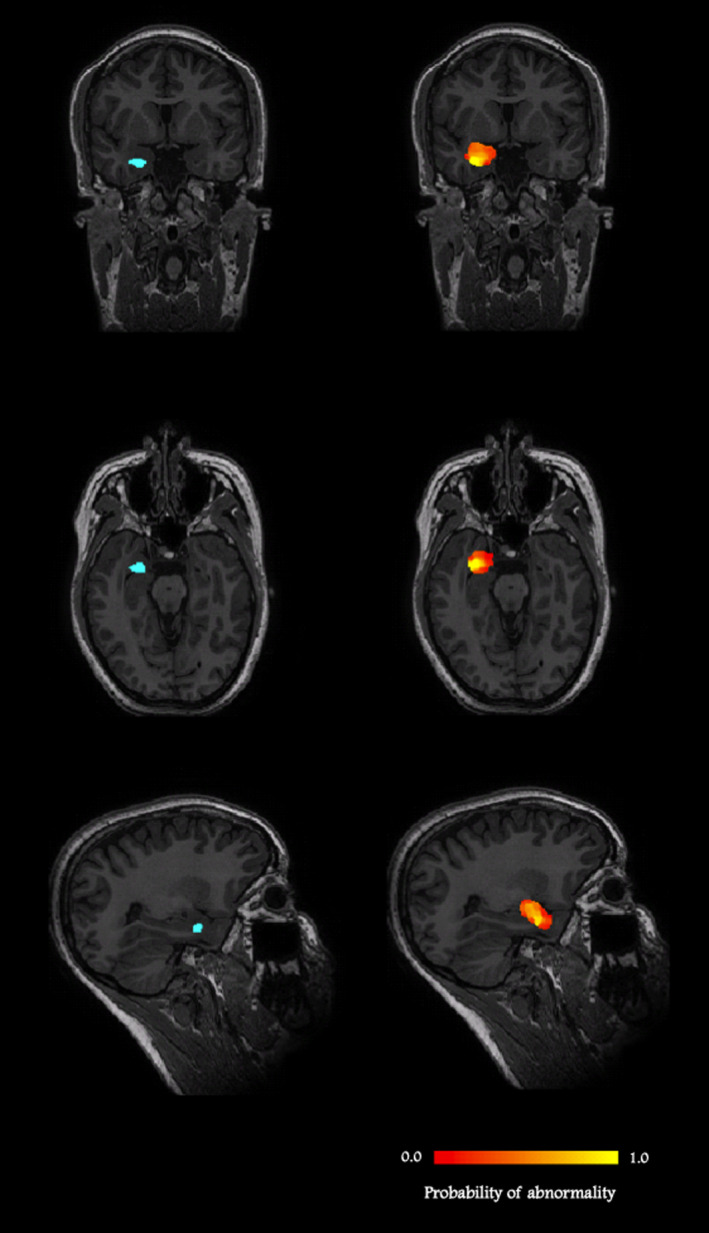
An MRI‐positive case: a 27‐year‐old female patient, with right amygdala dysembryoplastic neuroepithelial tumor. Images on the left show the manually drawn lesion mask in blue, whereas the images on the right show the probabilistic abnormality map detected by C2_vox_ in red (lowest probability) to yellow scale (highest probability). The lowest and highest probabilities of abnormality found in the lesional area detected in this case were 3% and 91%, respectively, with a mean over the lesion of 36%

In the MRI‐positive cases, the median DSC, measuring the degree of spatial overlap between the manually drawn lesion masks and the lesions detected by C2_vox_ was 0.59 (IQR: 0.37–0.78), measured using the leave‐one‐out cross‐validation scheme. The median over all MRI‐positive cases of the sensitivity and specificity of voxelwise lesion detection were 0.55 (IRQ: 0.21–0.81), and 0.9997 (IRQ: 0.9994–0.9999). An example case is shown in Figure 3. It is noted that the values of DSC should be read with the understanding that a value of 1.0 for DSC requires a perfect overlap between the detected lesional area and the manually drawn abnormality mask. The latter only reflects the extent of the abnormality that is visible on conventional MRI and does not necessarily correspond to the full extent of the epileptogenic lesion.

Grouped by pathology, the overall spatial agreements were 0.78 (IQR: 0.74–0.82) for hippocampal sclerosis, and 0.38 (IQR: 0.11–0.49) for other pathologies. For the latter group, there was no clear relationship between the pathology identified on radiological inspection and the DSC value.

A case with low spatial agreement is shown in Figure S1, which had a dice score coefficient of 0.2 or 20%. This is a 19‐year‐old female patient with blurring of the gray/white matter interface at the left middle temporal gyrus, in whom low probabilities of abnormality were detected both in the radiologically defined area of dysplasia and adjacent areas of the brain. The dysplastic lesion may have extended beyond that visible on conventional MRI, but no histology was available because the patient died from sudden unexpected death in epilepsy while awaiting surgery.

We also evaluated the value of the first‐stage classifier (C1_reg_) by withholding the output from it from C2_vox_ and observed an approximate decrease in DSC of 6% over all MRI‐positive cases. The corresponding decreases in sensitivity and specificity were 10% and 0.01%.

In comparison, the decreases in spatial agreement measured in terms of the dice score coefficient upon separately removing each modality as input to C2_vox_ were 0.6% for the T1‐weighted image, 2.1% for the FLAIR image, 1.4% for the NODDI NDI, 0.6% for DTI FA, 0.4% for DTI RD, and 0.8% for DTI AD (Figure S2). We note that the outputs of the first (C1_reg_) and second‐stage classifiers (C2_vox_) are amalgamations of the information contained in all the input modalities. We observed that the T1‐weighted image had a relatively small contribution to the output of C2_vox_ having already been used for structural assessment by C1_reg_.

Across all MRI‐positive cases, there was not a significant systematic difference between the lesion volumes measured using the manually drawn and the C2_vox_‐detected lesion masks (median volume: 7239 mm^3^ vs 7891 mm^3^, *p* = .74, 2‐tailed Mann‐Whitney *U* test).

No lesional areas were detected in any of the 62 control cases.

**FIGURE 4:**
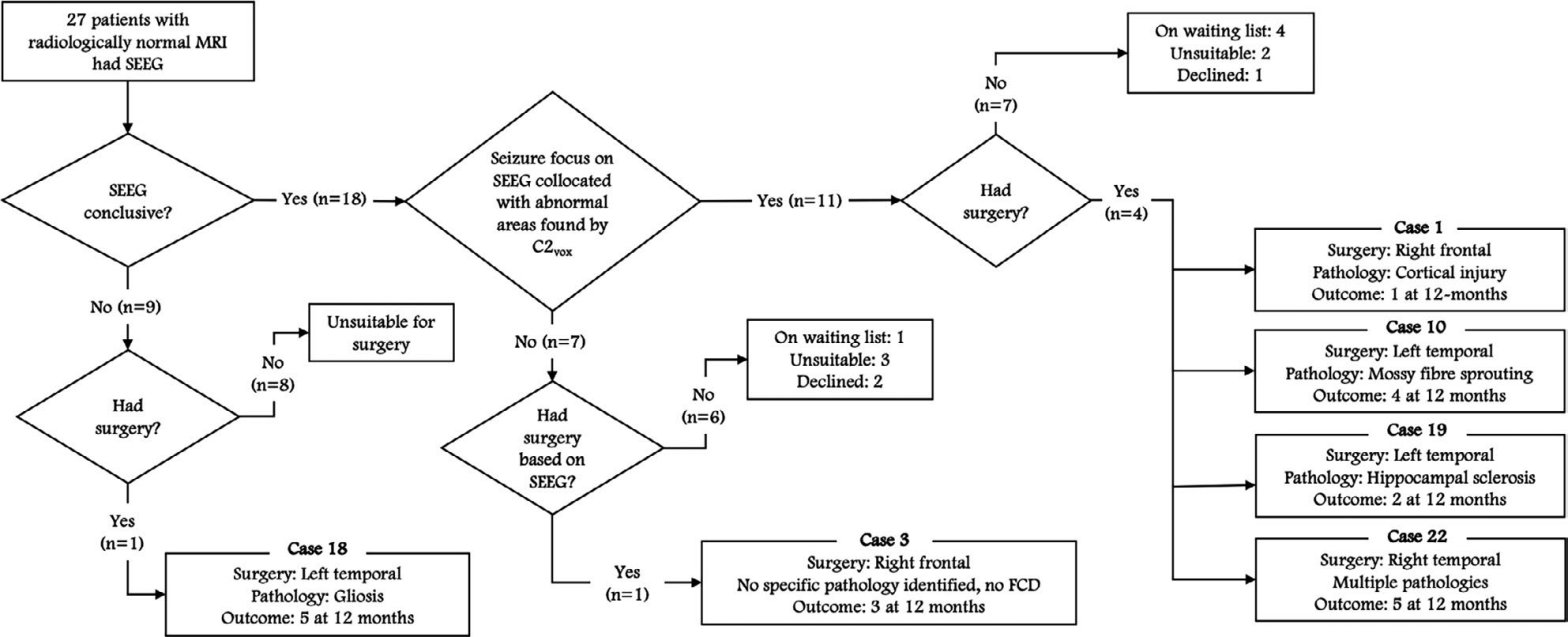
The agreement between SEEG and the abnormal areas detected by C2_vox_ along with surgical outcomes. SEEG was conclusive in 18 of 27 cases. In 11 of these 18 cases, a SoZ was found at SEEG, which collocated with the abnormal areas detected by C2_vox_

**FIGURE 5:**
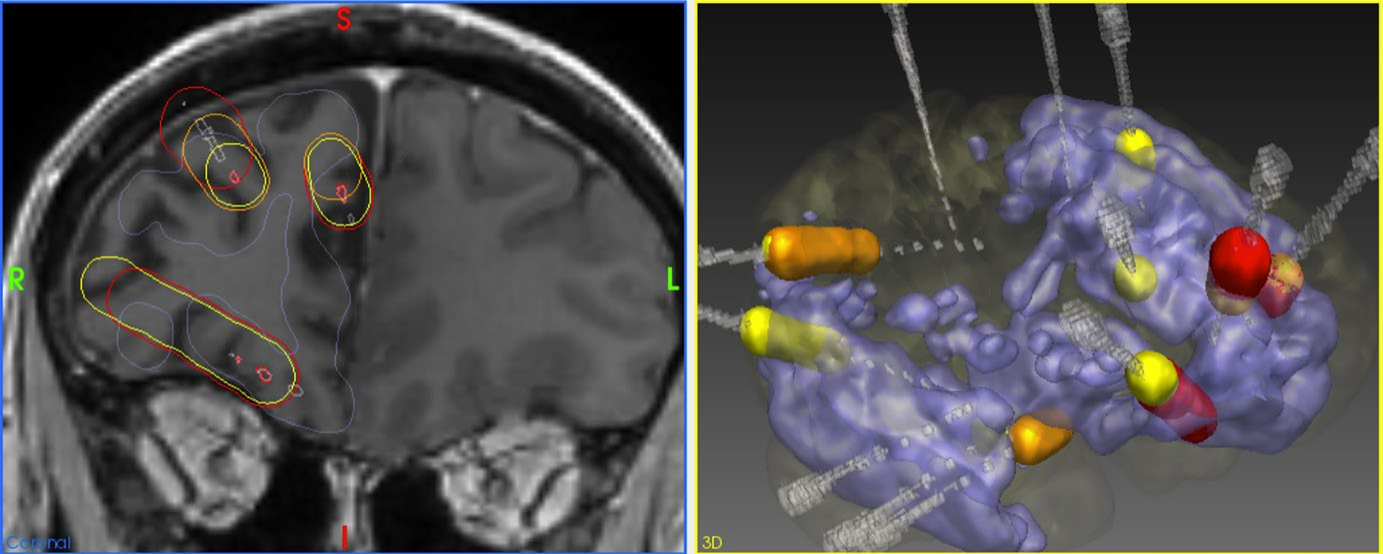
Structured comparison of the anatomical coordinates of abnormalities detected by C2_vox_ (blue), with the results of SEEG in an example case (case 1). SEEG electrodes are shown in gray, whereas the contacts that were active during the seizure onset, early propagation, and interictal activity are shown in red, orange, and yellow, respectively. The assessment in this case was that there was seizure onset, early seizure spread, and interictal discharges within the abnormal areas detected by C2_vox_ but there were also abnormal areas detected by C2_vox_ which were sampled at SEEG but not found to be involved in seizure onset

In the MRI negative cases, the SoZ was identified with SEEG in 18 (67%) of the 27 patients (Figure 4, Table S4). In 11 (61%) of these 18 patients, the SoZ found at SEEG was within the abnormal areas detected by C2_vox_ (Group A, SoZ detected). Of the four patients in this group who had surgery, one had a right temporal resection with an International League Against Epilepsy (ILAE) outcome of 5 at 12 months. The patient had multiple pathologies found on ex vivo inspection of the excised tissues (case 22, Table S4). Two other patients had left temporal resections with ILAE outcomes of 4 at 12 months, and 2 at 12 months, respectively (cases 10 and 19). In one of these cases, there were additional abnormal areas detected by C2_vox_, which were not sampled at SEEG, that could also have been involved in seizures (case 10). One patient had a right frontal resection with an ILAE outcome of 1 at 12 months (case 1). This was the only case in group A that had false positives, identified as additional abnormal areas found by C2_vox_ that were sampled at SEEG but did not give rise to seizures (Figure 5).

In the remaining 7 (39%) of the 18 of the cases for which SEEG was conclusive, the SoZ found at SEEG was not within the abnormal areas detected by C2_vox_ (Group B, SoZ not detected). However, in all seven cases, there was either early seizure propagation and/or interictal epileptiform discharges within the abnormal areas detected by C2_vox_ (cases 3, 5, 15, 16, and 25) and/or additional abnormal areas were found by C2_vox_ that were not sampled by the intracranial electrodes (cases 3, 9, and 12). Only one patient in this group had surgery (case 3). No specific pathology was identified on ex vivo inspection of the excised tissues for this patient, who had a right frontal resection. Their ILAE outcome was 3 at 12 months. This patient also had additional abnormal areas detected by C2_vox_, which were not sampled at SEEG.

In terms of false positives, there were abnormal areas found by C2_vox_ that were sampled by the SEEG electrodes but did not give rise to seizures in 6 (33%) of the 18 cases for which SEEG was conclusive. However, in all these cases, there was early seizure propagation and/or interictal epileptiform discharges found within the detected areas (cases 1, 3, 5, 15, 16, and 25; Table S4).

For the patients who had postsurgical outcome data available and had abnormalities found by C2_vox_ that were not sampled by the SEEG electrodes, the ILAE outcome was suboptimal (3 at 12 months and 4 at 12 months for cases 3 and 10, respectively).

SEEG was inconclusive in 9 (33%) of the 27 MRI negative cases (Group C, SoZ unknown). In one patient (case 18), ictal and interictal scalp video‐EEG was suggestive of left temporal onset, but no spontaneous seizures were captured on SEEG. Amygdala stimulation elicited a seizure with similar semiology and although not absolutely specific, the clinical consensus was a mesiotemporal onset for which left temporal resection was undertaken with an ILAE outcome at 12 months of 5. There were interictal epileptiform discharges during SEEG within the abnormal areas detected by C2_vox_ in this case.

A further patient had abnormal areas found by C2_vox_ that were not sampled at SEEG, which may have helped localize the epileptogenic zone (case 2, Table S4). The patient's semiology was olfactory/gustatory aura, suggesting limbic/temporal onset, and although the SoZ was not located at SEEG, ictal SPECT and scalp EEG both suggested a right hemispheric onset. There were two areas of abnormality found by C2_vox_ which were located at the right occipital pole and the left lateral orbital gyrus but they were not sampled by the SEEG electrodes. The electroclinical picture did not suggest a left orbitofrontal involvement but it is possible that the seizures started in the right occipital lobe, and propagated anteriorly, so an additional SEEG electrode placed in this proximity may have helped identify the SoZ.

Overall, there was early seizure propagation and/or interictal epileptiform discharges within the abnormal areas found by C2_vox_ in six (67%) of the nine cases for whom SEEG was inconclusive.

## 4 DISCUSSION

Medically refractory focal epilepsy presenting with radiologically normal MRI is a major clinical challenge.^2^ A computational method for detecting covert lesions that uses multimodal MRI data may help meet this challenge. We present such a method that intends to detect the diverse range of pathologies that can result in medically refractory focal epilepsy. In addition to the macroscopic abnormalities that may be visible on conventional radiological sequences, these also include microscopic abnormalities, which may only be apparent in altered diffusion or magnetic susceptibility properties of tissues, and abnormalities that are too subtle to be noticed on unassisted visual reading.

In the patients with medically refractory focal epilepsy and radiologically normal MRI with a SoZ identified at SEEG, the majority (11/18, or 61%) had a SoZ within the abnormal areas detected by our method. In those where this was not the case, most (5/7, or 71%) demonstrated early seizure propagation and/or interictal epileptiform discharges within the abnormal areas found by our method, suggesting that the approach offers promise. This was also true for patients in whom a SoZ was not located at SEEG where this value was 6/9 or 66%.

Our method uses a two‐stage detection scheme. Our C1_reg_ assesses each parcellated region of the brain separately and produces a probabilistic, regional brain abnormality map. The second‐stage classifier (C2_vox_) then integrates this regional brain abnormality map, alongside the available MRI data, into a voxelwise, probabilistic map of brain abnormalities. The rationale behind this approach is that epilepsy can result not only from an abnormality of a specific brain region (eg, hippocampal sclerosis) but also from abnormalities that span across more than one area of the brain (eg, focal cortical dysplasia). Although both types of abnormalities can be detected in a voxelwise manner, our results support the hypothesis that regional abnormalities may be best detected with input from a prior, regional assessment: withholding of the output from C1_reg_ as an input to C2_vox_ resulted in a decrease in DSC of ~6% for the MRI‐positive cases.

Our comparison of the computationally detected abnormalities with SEEG data enables us to assess whether these areas give rise to epileptic seizures, and/or are involved in early seizure propagation, and interictal epileptiform discharges.

Our study has limitations. The first of these is the limited number of MRI‐positive lesional cases (*n* = 42) we used to train our system. However, despite this limitation, we observed a promising degree of covert epileptogenic lesion detection in radiologically normal, medically refractory focal epilepsy. We continue to enroll patients in our program, and with a developing imaging database comprising a diverse range of underlying pathologies, we hope to increase the strength of our technique. A growing imaging database will also enable us to test the hypothesis that the underlying pathology (eg, focal cortical dysplasia vs gliosis) may also be inferred from multimodal MRI data.

The second limitation of our study is the small number of surgical outcomes that were available. This was due partly to the high attrition rate (16/27, or 59%) following SEEG, and partly due to the ongoing severe acute respiratory syndrome coronavirus 2 (SARS‐CoV‐2) pandemic, which meant that some surgeries had to be put on hold (*N* = 5). Future evaluation would benefit from a larger number of postoperative outcomes, and a prospective assessment of the utility of the method in planning SEEG coverage.

Our method has not yet been applied to data acquired at other centers. However, it is primed for combatting batch effects such as the effect of the scanner through our use of standardized processing pipelines resulting in quantitative parametric maps (eg, DTI FA, NODDI NDI), and the normalization of raw image volumes (eg, the T1‐weighted image). Furthermore, the flexibility offered by machine learning makes our method capable of modeling‐out batch effects when it is expanded to a multi‐center setting by means of utilizing batch‐related parameters (eg, scanner manufacturer and model) as additional variables.

Further development of our imaging database is expected to reduce the number of cases in whom the seizure focus found at SEEG does not collocate with the abnormal areas found by C2_vox_ (7/18 or 39%) and to allow us to quantitatively assess whether agreement between the two improves postsurgical outcomes. In the present study, we have found qualitative evidence suggesting that in cases in whom postsurgical outcome data are available, and there are additional abnormalities found by C2_vox_,which are not sampled at SEEG, the outcome may be suboptimal. Further work will determine whether taking into account the abnormalities detected by C2_vox_ when planning SEEG can increase the number of occasions in which curative surgery can be offered to patients and improve postsurgical outcomes for those who undergo surgery.

In our study, SEEG was inconclusive in 9 of 27 patients (33%). In one patient this was due to the absence of any spontaneous seizures during SEEG, whereas a clear SoZ could not be identified in the remaining eight patients. These cases remain a challenge. However, as the imaging database grows, covert abnormalities in such cases may become detectable. Also of note is that one of these patients had abnormal areas found by C2_vox_, which were not sampled at SEEG, and the consideration of the computationally detected abnormalities might have helped refine the SEEG sampling strategy and identify the SoZ.

Because contemporary computational methods may offer the possibility of delineating the full extent of lesions extending beyond what is visible on conventional MRI, the additional information obtained from such methods may also help in the pre‐surgical evaluation of MRI‐positive cases. However, the focus of the present study was the 27 cases with no visible MRI lesions where the 42 cases with visible lesions were used as a training set to develop the computational method.

A comparison to existing published computational methods would be welcome but challenging due to differences in patient cohorts and assessment criteria. Our approach aims to detect any type of lesion, whereas other voxel‐based techniques^32^ and surface‐based approaches^9^ are designed only to detect focal cortical dysplasia. Furthermore, inclusion criteria differ as these studies include subjects whose lesions were detected visually in 65% of cases^32^ or all cases with texture map post‐processing techniques.^9^

## 5 CONCLUSION

Computational analysis of multimodal MRI data may help in identifying epileptogenic lesions in medically refractory focal epilepsy, and radiologically normal MRI. Comparison with the areas of seizure onset, early propagation and interictal epileptiform discharges found with SEEG helps validate the method used for the detection of the covert lesions. Further work, including the building of a large database of MRI‐positive lesional cases, is expected to improve the value of the technique and determine whether consideration of the computationally detected lesions in intracranial EEG and surgical planning can increase the number of patients that can be offered surgery, as well as improve post‐surgical outcomes.

## CONFLICT OF INTEREST

GJB receives honoraria for teaching from GE Healthcare. The other authors have no disclosures or conflicts of interest to report. We confirm that we have read the Journal's position on issues involved in ethical publication and affirm that this report is consistent with those guidelines.

## Supporting information

Supplementary Material

## Data Availability

The lesion‐detection method described in this study will be made freely available to the research community on GitHub at the short link https://bit.ly/35Y0GOC. The data sets used during development are not publicly available due to ethical restrictions.

## References

[1] Leeman‐Markowski B. Review of MRI‐negative epilepsy. JAMA Neurol. 2016;73(11):1377.

[2] Bernasconi A, Bernasconi N, Bernhardt BC, Schrader D. Advances in MRI for “cryptogenic” epilepsies. Nat Rev Neurol. 2011;7(2):99–108. 21243016 10.1038/nrneurol.2010.199

[3] Bennett OF, Kanber B, Hoskote C, Cardoso MJ, Ourselin S, Duncan JS, et al. Learning to see the invisible: a data‐driven approach to finding the underlying patterns of abnormality in visually normal brain magnetic resonance images in patients with temporal lobe epilepsy. Epilepsia. 2019;60(12):2499–507. 31691273 10.1111/epi.16380 PMC6972547

[4] Keihaninejad S, Heckemann RA, Gousias IS, Hajnal JV, Duncan JS, Aljabar P, et al. Classification and lateralization of temporal lobe epilepsies with and without hippocampal atrophy based on whole‐brain automatic MRI segmentation. PloS one [Internet]. 2012;7(4):e33096. 10.1371/journal.pone.0033096 PMC3327701 22523539

[5] Huppertz H‐J, Grimm C, Fauser S, Kassubek J, Mader I, Hochmuth A, et al. Enhanced visualization of blurred gray‐white matter junctions in focal cortical dysplasia by voxel‐based 3D MRI analysis. Epilepsy Res. 2005;67(1–2):35–50. 16171974 10.1016/j.eplepsyres.2005.07.009

[6] Azami ME, Hammers A, Jung J, Costes N, Bouet R, Lartizien C. Detection of lesions underlying intractable epilepsy on T1‐weighted MRI as an outlier detection problem. PLoS One. 2016;11(9):e0161498. 27603778 10.1371/journal.pone.0161498 PMC5015774

[7] Ahmed B, Thesen T, Blackmon KE, Kuzniekcy R, Devinsky O, Brodley CE. Decrypting “cryptogenic” epilepsy: semi‐supervised hierarchical conditional random fields for detecting cortical lesions in MRI‐negative patients. J Machine Learn Res. 2016;17(112):1–30.

[8] Jin B, Krishnan B, Adler S, Wagstyl K, Hu W, Jones S, et al. Automated detection of focal cortical dysplasia type II with surface‐based magnetic resonance imaging postprocessing and machine learning. Epilepsia. 2018;59(5):982–92. 29637549 10.1111/epi.14064 PMC5934310

[9] Hong S‐J, Kim H, Schrader D, Bernasconi N, Bernhardt BC, Bernasconi A. Automated detection of cortical dysplasia type II in MRI‐negative epilepsy. Neurology. 2014;83(1):48–55. 24898923 10.1212/WNL.0000000000000543 PMC4114179

[10] Alaverdyan Z, Jung J, Bouet R, Lartizien C. Regularized siamese neural network for unsupervised outlier detection on brain multiparametric magnetic resonance imaging: application to epilepsy lesion screening. Med Image Anal. 2020;60:101618. 31841950 10.1016/j.media.2019.101618

[11] Gill RS, Hong S‐J, Fadaie F, Caldairou B, Bernhardt BC, Barba C, et al. Deep convolutional networks for automated detection of epileptogenic brain malformations. In: Frangi AF, Schnabel JA, Davatzikos C, Alberola‐López C, Fichtinger G, editors. Medical image computing and computer assisted intervention – MICCAI 2018. Cham: Springer International Publishing; 2018. p. 490–7.(Lecture Notes in Computer Science).

[12] Salmenpera TM, Symms MR, Rugg‐Gunn FJ, Boulby PA, Free SL, Barker GJ, et al. Evaluation of quantitative magnetic resonance imaging contrasts in MRI‐negative refractory focal epilepsy. Epilepsia. 2007;48(2):229–37. 17295615 10.1111/j.1528-1167.2007.00918.x

[13] Kanber B, Duncan JS, Rodionov R, Chowdhury FA, Winston GP. Validation of computational lesion detection methods in magnetic resonance imaging–negative, focal epilepsy. Epilepsia. 2020;61(4):828–30. 32100283 10.1111/epi.16461 PMC7618163

[14] Kini LG, Gee JC, Litt B. Computational analysis in epilepsy neuroimaging: a survey of features and methods. Neuroimage Clin. 2016;11:515–29. 27114900 10.1016/j.nicl.2016.02.013 PMC4833048

[15] Mo J‐J, Zhang J‐G, Li W‐L, Chen C, Zhou N‐J, Hu W‐H, et al. Clinical value of machine learning in the automated detection of focal cortical dysplasia using quantitative multimodal surface‐based features. Front Neurosci. 2019;12:1008. 30686974 10.3389/fnins.2018.01008 PMC6336916

[16] Jenkinson M, Beckmann CF, Behrens TEJ, Woolrich MW, Smith SM. FSL. NeuroImage. 2012;62(2):782–90. 21979382 10.1016/j.neuroimage.2011.09.015

[17] Zhang H, Schneider T, Wheeler‐Kingshott CA, Alexander DC. NODDI: Practical in vivo neurite orientation dispersion and density imaging of the human brain. NeuroImage. 2012;61(4):1000–16. 22484410 10.1016/j.neuroimage.2012.03.072

[18] Deoni SCL, Peters TM, Rutt BK. High‐resolution T1 and T2 mapping of the brain in a clinically acceptable time with DESPOT1 and DESPOT2. Magn Reson Med. 2005;53(1):237–41. 15690526 10.1002/mrm.20314

[19] Deoni SCL, Rutt BK, Peters TM. Rapid combined T1 and T2 mapping using gradient recalled acquisition in the steady state. Magn Reson Med. 2003;49(3):515–26. 12594755 10.1002/mrm.10407

[20] Wood TC. QUIT: QUantitative Imaging Tools. J Open Source Softw. 2018;3(26):656.

[21] Ke G, Meng Q, Finley T, Wang T, Chen W, Ma W, et al. LightGBM: a highly efficient gradient boosting decision tree. In: Guyon I, Luxburg UV, Bengio S, Wallach H, Fergus R, Vishwanathan S, et al., Advances in neural information processing systems 30 [Internet]. Red Hook, NY: Curran Associates, Inc.; 2017. p. 3146–54.Available from: http://papers.nips.cc/paper/6907‐lightgbm‐a‐highly‐efficient‐gradient‐boosting‐decision‐tree.pdf

[22] Chen G, Li Q, Shi F, Rekik I, Pan Z. RFDCR: automated brain lesion segmentation using cascaded random forests with dense conditional random fields. NeuroImage. 2020;211:116620. 32057997 10.1016/j.neuroimage.2020.116620

[23] Wang L, Gao Y, Shi F, Li G, Gilmore JH, Lin W, et al. LINKS: learning‐based multi‐source IntegratioN frameworK for segmentation of infant brain images. NeuroImage. 2015;108:160–72. 25541188 10.1016/j.neuroimage.2014.12.042 PMC4323750

[24] Geremia E, Clatz O, Menze BH, Konukoglu E, Criminisi A, Ayache N. Spatial decision forests for MS lesion segmentation in multi‐channel magnetic resonance images. NeuroImage. 2011;57(2):378–90. 21497655 10.1016/j.neuroimage.2011.03.080

[25] Song B, Chou C‐R, Chen X, Huang A, Liu M‐C. Anatomy‐guided brain tumor segmentation and classification. In: Crimi A, Menze B, Maier O, Reyes M, Winzeck S, Handels H, editors. Brainlesion: glioma, multiple sclerosis, stroke and traumatic brain injuries. Cham: Springer International Publishing; 2016. p. 162–70.(Lecture Notes in Computer Science).

[26] Mitra J, Bourgeat P, Fripp J, Ghose S, Rose S, Salvado O, et al. Lesion segmentation from multimodal MRI using random forest following ischemic stroke. NeuroImage. 2014;98:324–35. 24793830 10.1016/j.neuroimage.2014.04.056

[27] Pustina D, Coslett HB, Turkeltaub PE, Tustison N, Schwartz MF, Avants B. Automated segmentation of chronic stroke lesions using LINDA: lesion identification with neighborhood data analysis. Hum Brain Mapp. 2016;37(4):1405–21. 26756101 10.1002/hbm.23110 PMC4783237

[28] Modat M, Ridgway GR, Taylor ZA, Lehmann M, Barnes J, Hawkes DJ, et al. Fast free‐form deformation using graphics processing units. Comput Methods Programs Biomed. 2010;98(3):278–84. 19818524 10.1016/j.cmpb.2009.09.002

[29] Cardoso MJ, Modat M, Wolz R, Melbourne A, Cash D, Rueckert D, et al. Geodesic information flows: spatially‐variant graphs and their application to segmentation and fusion. IEEE Trans Med Imaging. 2015;34(9):1976–88. 25879909 10.1109/TMI.2015.2418298

[30] Avants BB, Tustison N, Song G. Advanced normalization tools (ANTS). Insight J. 2009;1:1–29.

[31] Zombori G, Rodionov R, Nowell M, Zuluaga MA, Clarkson MJ, Micallef C, et al. A computer assisted planning system for the placement of sEEG electrodes in the treatment of epilepsy. In: Stoyanov D, Collins DL, Sakuma I, Abolmaesumi P, Jannin P, editors. Information processing in computer‐assisted interventions. Cham: Springer International Publishing; 2014. p. 118–27.(Lecture Notes in Computer Science).

[32] Wagner J, Weber B, Urbach H, Elger CE, Huppertz H‐J. Morphometric MRI analysis improves detection of focal cortical dysplasia type II. Brain. 2011;134(10):2844–54. 21893591 10.1093/brain/awr204

