# Supplementary material for: Detection of covert lesions in focal epilepsy using computational analysis of multimodal magnetic resonance imaging data
Source: Epilepsia. 2021 Feb 10;62(3):807–16. doi: 10.1111/epi.16836 (PMC8436754; doi:10.1111/epi.16836)

**Supplementary Table 1** - Basic clinical and demographic details of the MRI negative patients.

| **Case** | **Gender** | **Age (years)** | **Disease duration (years)** | **Seizure semiology** | **EEG findings** | **Neuropsychology** |
| --- | --- | --- | --- | --- | --- | --- |
| 1 | Female | 34 | 7 | Dialeptic -> automotor (no lateralising) | Right inferior frontotemporal (ictal), bitemporal (interictal) | Frontal (no side) |
| 2 | Male | 36 | 32 | Dialeptic -> automotor (no lateralising) | Right hemisphere (ictal), R > L temporal (interictal) | No lateralising/localising |
| 3 | Male | 25 | 19 | Autonomic -> left versive (right frontal) | Right frontocentral (interictal, ictal) | Frontal (no side) [prev ND temp] |
| 4 | Male | 21 | 13 | Left hemisphere | Left frontocentroparietal (interictal, ictal) [also right temporal interictal] | Frontal (no side) |
| 5 | Male | 22 | 11 | Multiple, some right hemisphere | Bilateral posterior quadrant (ictal), bilateral f-c and t-o (interictal) | Widespread |
| 6 | Male | 41 | 27 | Left hemisphere (frontal, temporal) | Left paracentral (ictal), bilateral frontotemporal (interictal) | Left temporal |
| 7 | Female | 36 | 29 | Multiple, some right hemisphere | Right frontocentral (ictal), right anterior frontal (interictal) | Left frontotemporal |
| 8 | Female | 40 | 22 | Aura>dialeptic/automotor->GTCS | Right>left temporal (interictal), right temporal (ictal) | Bilateral involvement |
| 9 | Male | 34 | 18 | Multiple, some right hemisphere (left arm) | Right frontotemporal (interictal), vertex right>left (ictal) | R>L frontotemporal |
| 10 | Male | 21 | 6 | Autonomic->dialeptic (?left, aphasia) | Left frontotemporal (interictal, ictal) | Left temporal |
| 11 | Female | 31 | 27 | Multiple (no lateralising) | Widespread (interictal), bihemispheric left>right (ictal) | No lateralising/localising |
| 12 | Female | 24 | 10 | Automotor (left) | Left temporal/paracentral (ictal), nil (interictal) | No lateralising/localising |
| 13 | Male | 24 | 9 | Multiple (left) | Left frontocentral (interictal), also temporal; non-localised (ictal) | Widespread dominant |
| 14 | Male | 23 | 18 | Autonomic->automotor (right) | Right temporal (interictal), right temporal+frontocentral (ictal) | No lateralising/localising |
| 15 | Male | 31 | 15 | Multiple (left frontal) | Bifrontal R>L (interictal, ictal) | Left frontal (+temporal) |
| 16 | Male | 40 | 33 | Multiple (no lateralising) | R>L frontal (interictal), non-localising? R frontocentral (ictal) | No lateralising/localising |
| 17 | Female | 38 | 32 | Abdominal>autonomotor (right) | Right anterior temporal (interictal, ictal) | Non-dominant temporal |
| 18 | Male | 38 | 18 | Aura>automotor>GTCS (left) | Left temporal (interictal, ictal) | Dominant temporal |
| 19 | Female | 41 | 38 | Abdominal>automotor+/-GTCS | Right temporal (interictal), left temporal + NEAD (ictal) | Frontal (no side) |
| 20 | Female | 41 | 16 | Somatosensory/other>automotor (left) | Left frontocentral/temporal (interictal), left frontocentral (ictal) | No lateralising/localising |
| 21 | Male | 47 | 25 | Right visual>somatosensory>GTCS (left) | Left temporal (interictal), left centroparietal-occipital (ictal) | Dominant fronto-temporal |
| 22 | Male | 20 | 6 | Psychic>dialeptic, psychic>hyperkinetic (right) | Right anterior temporal (interictal, ictal) | No lateralising/localising |
| 23 | Male | 35 | 9 | Psychic/autonomic>automotor, dialeptic/automotor (?right) | Left frontotemporal (interictal), left temporal or right hemisphere (ictal) | Left temporal |
| 24 | Female | 35 | 34 | Psychic>automotor (left), also FBTCS | Left temporal (interictal), left anterior temporal (ictal) | Left temporal |
| 25 | Female | 41 | 16 | Psychic>dialeptic>automotor (no lateralising) | Left>right temporal (interictal), left frontotemporal (ictal) | Left temporal |
| 26 | Male | 30 | 22 | Complex motor (left) | Left>right temporal (interictal), left temporal (ictal) | Left frontotemporal |
| 27 | Male | 36 | 11 | Automotor (not lateralising) | Left>right frontocentrotemporal (interictal), non-localising (ictal) | Left temporal |

**Supplementary Table 2** - The magnetic resonance imaging (MRI) protocol included clinical and research sequences, and their respective derived measures. The images were obtained using a 3T GE MR750 scanner (General Electric, Waukesha, WI, USA) using standard imaging gradients. Raw image volumes (e.g. 3D T1, 3D FLAIR) were intensity normalised to have a zero mean and unit standard deviation.

| **Sequence and parameters** | **MRI scans and derived imaging volumes** |
| --- | --- |
| Fast spoiled gradient recalled echo (FSPGR); TE/TR/TI: 3.1/7.4/400 ms | 3D T1-weighted  Brain parcellation obtained using GIF^29^.  Cortical thickness map obtained using ANTS^30^. |
| Fast spin echo with variable flip-angle readout; TE/TR/TI: 137/6200/1882 ms | 3D FLAIR |
| 3D susceptibility-weighted angiography; TE: 13, 17.9, 22.7, 27.6, 32.5 ms; TR: 37.1 ms | T2* susceptibility-weighted angiography (SWAN) |
| Multi-shell diffusion-weighted 2mm isotropic single-shot spin echo sequence; TR/TE: 7600/74.1 ms; ∂/Δ = 21.5/35.9 ms.  A total of 115 volumes are acquired with 11, 8, 32, and 64 gradient directions at b-values of 0, 300, 700, and 2500 s/mm^2^ respectively, as well as a single b=0 image with reverse phase-encoding to allow distortion correction. | Map of fractional anisotropy (DTI FA)^16^  Map of radial diffusivity (DTI RD)^16^  Map of axial diffusivity (DTI AD)^16^  Map of neurite density index (NODDI NDI)^17^ |
| Spoiled gradient-recalled echo; flip angles: 3, 4, 5, 6, 7, 9, 13, 18 degrees; TR/TE: 8.3/2.5 ms; inversion-recovery spoiled gradient-recalled echo; flip angle: 5 degrees; TR/TE/TI: 8.3/2.5/450 ms; balanced steady-state free precession; flip angles: 12, 16, 21, 27, 33, 40, 51, 68 degrees (each with two phase cycling angles of 0 and 180 degrees); TR/TE: 4.4/2.2 ms | Driven equilibrium single pulse observation of T1/T2 (DESPOT)^18-20^  Map of T1 relaxation (DESPOT T1)  Map of proton density (DESPOT PD) |

**Supplementary Table 3** – Regional brain properties used by C1_reg_. All except the normalised volume were measured for every available MRI contrast (e.g. 3D T1, 3D FLAIR) and derived map (e.g. DTI AD, NODDI NDI, DESPOT T1).

| **Property** | **Description** |
| --- | --- |
| Normalised volume | Volume of the region (e.g. left hippocampus) normalised by the total intracranial volume. |
| Mean signal | Mean signal intensity within the region (e.g. mean FLAIR signal and mean radial diffusivity). |
| Signal coefficient of variation | Standard deviation of the signal intensity within the region divided by the mean signal intensity within the region. |

**Supplementary Table 4** - Structured comparison of the abnormal areas detected by C2_vox_ with the results of stereoelectroencephalography (SEEG), along with reported surgical outcomes, and the pathological assessment of excised tissues. SEEG was conclusive in 18/27 (67%) cases. In 11 (61%) of these 18 cases, a seizure onset zone (SoZ) was found at SEEG which collocated with an abnormal area detected by C2_vox_. In 9 (35%) of the 26 cases where there was a seizure at SEEG, there was early seizure propagation within the abnormal areas detected by C2_vox_. In 20/27 (74%) cases, there was interictal epileptiform discharges within the abnormal areas found by C2_vox_. In 6 (22%) of the 27 cases, there were abnormal areas found by C2_vox_ that were not sampled by the SEEG electrodes. In 6 (33%) of the 18 cases for which SEEG was conclusive, there was an abnormal area found by C2_vox_ that was sampled by the SEEG electrodes but did not give rise to seizures. Abbreviations: focal cortical dysplasia (FCD); hippocampal sclerosis (HS); malformations of cortical development (MCD).

| **Case** | **SEEG conclusive** | **SoZ in abnormal area** | **Early seizure propagation within abnormal area** | **Interictal epileptiform discharges within abnormal area** | **Abnormal area not sampled** | **Sampled abnormal area not give rise to seizures** | **Surgery** | **Pathology** | **Outcome (ILAE scale)** |
| --- | --- | --- | --- | --- | --- | --- | --- | --- | --- |
| 1 | yes | yes | yes | yes | no | yes | Right frontal resection | Chronic, patchy cortical destructive process (+FCD IIId) | 1 at 12 months |
| 4 | yes | yes | yes | yes | yes | no | Unsuitable for surgery |  |  |
| 10 | yes | yes | no | yes | yes | no | Left temporal resection | No evidence of HS but incomplete specimen, mild mossy fibre sprouting, no evidence of FCD | 4 at 12 months |
| 14 | yes | yes | no | yes | no | no | Patient declined surgery |  |  |
| 17 | yes | yes | yes | yes | no | no | On surgery waiting list |  |  |
| 19 | yes | yes | no | yes | no | no | Left temporal resection | Hippocampal sclerosis, type 3 | 2 at 12 months |
| 20 | yes | yes | yes | yes | no | no | On surgery waiting list |  |  |
| 22 | yes | yes | no | no | no | no | Right temporal resection | Hippocampal gliosis, small periventricular heterotopia and mild MCD type II | 5 at 12 months |
| 23 | yes | yes | no | yes | no | no | Unsuitable for surgery |  |  |
| 24 | yes | yes | no | yes | no | no | On surgery waiting list |  |  |
| 26 | yes | yes | no | yes | no | no | On surgery waiting list |  |  |
| 3 | yes | no | no | yes | yes | yes | Right frontal resection | No specific pathology identified (no cortical dysplasia) | 3 at 12 months |
| 5 | yes | no | no | yes | no | yes | Unsuitable for surgery |  |  |
| 9 | yes | no | no | no | yes | no | Unsuitable for surgery |  |  |
| 12 | yes | no | no | no | yes | no | Unsuitable for surgery |  |  |
| 15 | yes | no | no | yes | no | yes | Patient declined surgery |  |  |
| 16 | yes | no | yes | yes | no | yes | Patient declined surgery |  |  |
| 25 | yes | no | yes | no | no | yes | On surgery waiting list |  |  |
| 2 | no | SoZ not localised | no | no | yes | SoZ not localised | Unsuitable for surgery |  |  |
| 6 | no | SoZ not localised | no | yes | no | SoZ not localised | Unsuitable for surgery |  |  |
| 7 | no | SoZ not localised | no | no | no | SoZ not localised | Unsuitable for surgery |  |  |
| 8 | no | SoZ not localised | yes | yes | no | SoZ not localised | Unsuitable for surgery |  |  |
| 11 | no | SoZ not localised | no | yes | no | SoZ not localised | Unsuitable for surgery |  |  |
| 13 | no | SoZ not localised | yes | yes | no | SoZ not localised | Unsuitable for surgery |  |  |
| 21 | no | SoZ not localised | no | no | no | SoZ not localised | Unsuitable for surgery |  |  |
| 27 | no | SoZ not localised | yes | yes | no | SoZ not localised | Unsuitable for surgery |  |  |
| 18 | no | no seizures during SEEG | no seizures during SEEG | yes | no | no seizures during SEEG | Left temporal resection | Gliosis in left hippocampus and amygdala | 5 at 12 months |

**Supplementary Figure 1** - An MRI positive case with low spatial agreement. This is a 19-year-old female patient with blurring of the grey/white matter interface at the left middle temporal gyrus found on radiological inspection. First and third images show the manually drawn lesion mask in blue, while the second and fourth images show the probabilistic abnormality map detected by C2_vox_ in red (lowest probability) to yellow scale (highest probability).


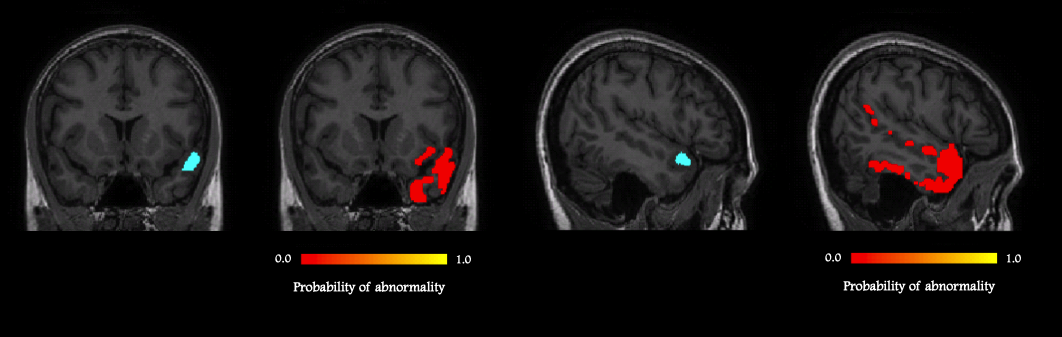


**Supplementary Figure 2** - The value of each modality as measured by the drop in the value of the dice score coefficient (DSC) over all MRI positive cases upon the separate removal of each modality as an input to the voxelwise classifier C2_vox_. The T1-weighted image appeared to be relatively less informative having already been used for structural assessment by the first-stage classifier C1_reg_.


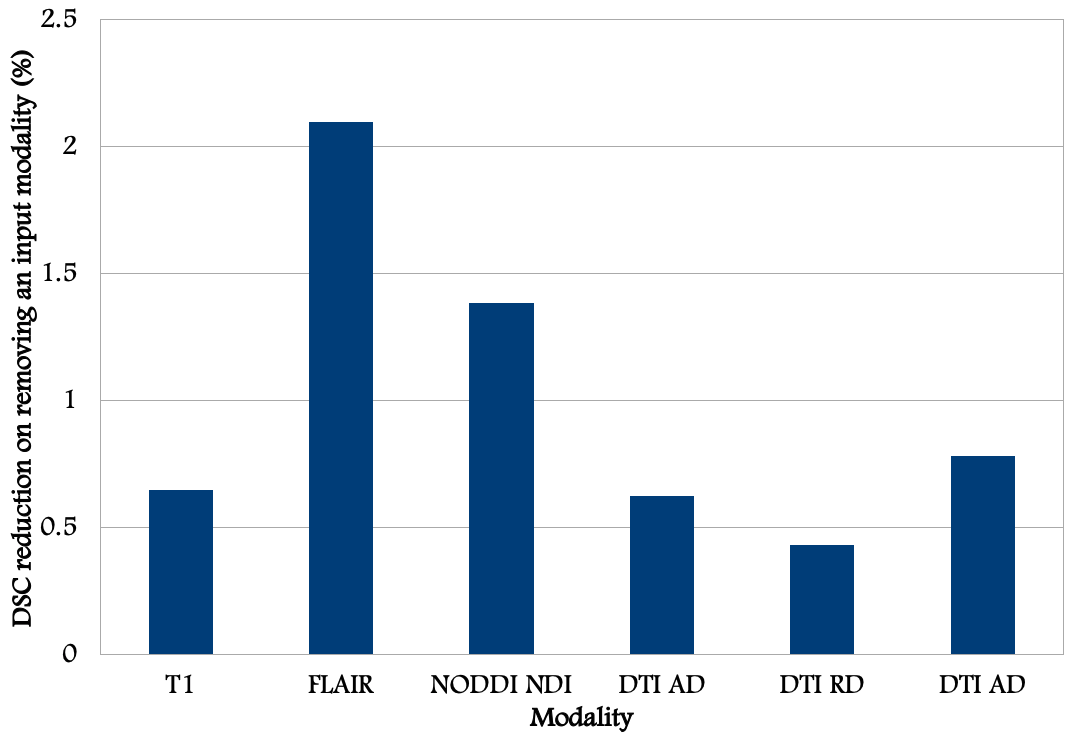

Supplement: Supplementary file 1 — Supplementary Material [file EPI-62-807-s001.docx]
